# Palermo Prison Hospital

**Published:** 1831-03

**Authors:** 


### HOSPITAL REPORTS.

### PALERMO PRISON HOSPITAL.

*Internal Strangulation, formed by a Part of the Transverse Arch of the Colon, which passed through an Opening in the Diaphragm.* By Dr. Larussac.†

A man, named Giovanni Mangiapane, twenty-seven years of age, was admitted January 22d, 1830. He complained of violent pains in the epigastric region, to which he had been, more or less, subject since his infancy: his bowels had not been open for several days; pulse full. He was ordered castor oil, and emollient cataplasms to the belly; blood was also taken from the arm; the pains, however, continued increasing, and extended into the interior of the chest. Fever, and obstinate vomiting, took place on the fourth day; tongue dry: the same treatment was continued; in addition to which, leeches were applied to the belly. As the vomiting continued, and the abdomen became tympanitic, cold fomentations and iced drinks were ordered; but without relief. Stercoraceous vomiting, violent colic, increased tympany, small and rapid pulse, now left no doubt as to the nature of the disease.

On the ninth day, the countenance of the patient, and the general symptoms, indicated approaching death. A tobacco enema was administered, and, as copious stools followed, some hope was still entertained: but the vomiting again returned; crude mercury was given, without avail; and, on the 4th of February, the patient expired in great torment.

*Dissection.* The most attentive examination failed to detect any

† Giornale Patologico-Medico-Chirurgico di Palermo.

invagination of the intestines, but they were highly inflamed throughout their whole extent to the rectum. The transverse arch of the colon was displaced: it occupied the cavity of the thorax, and was soft and brown, from having become gangrenous; it was enveloped by the peritoneum, which adhered to the opening through which it had passed. This opening was of an oval shape, and situated at the left side of the diaphragm, and quite independent of the apertures which give passage to the vena cava and œsophagus: it was large enough for a hen's egg to pass through; on its upper part was a circular fold, which formed a kind of valve. The aperture could not be regarded as the effect of disease, as the edges of it were round, and formed by a fold of the aponeurotic portion of the diaphragm, and the fibres of the muscles terminated round them in a very regular manner.

